# Dissecting weed adaptation: Fitness and trait correlations in herbicide‐resistant *Alopecurus myosuroides*

**DOI:** 10.1002/ps.6930

**Published:** 2022-05-09

**Authors:** David Comont, Dana R MacGregor, Laura Crook, Richard Hull, Lieselot Nguyen, Robert P Freckleton, Dylan Z Childs, Paul Neve

**Affiliations:** ^1^ Department of Biointeractions and Crop Protection Rothamsted Research, Harpenden Hertfordshire UK; ^2^ Department of Biosciences University of Durham Durham UK; ^3^ Department of Animal and Plant Sciences University of Sheffield South Yorkshire UK; ^4^ Department of Plant and Environmental Sciences, Section for Crop Sciences University of Copenhagen Taastrup Denmark

**Keywords:** quantitative genetics, non‐target‐site, herbicide resistance, fitness, evolutionary potential

## Abstract

**BACKGROUND:**

Unravelling the genetic architecture of non‐target‐site resistance (NTSR) traits in weed populations can inform questions about the inheritance, trade‐offs and fitness costs associated with these traits. Classical quantitative genetics approaches allow study of the genetic architecture of polygenic traits even where the genetic basis of adaptation remains unknown. These approaches have the potential to overcome some of the limitations of previous studies into the genetics and fitness of NTSR.

**RESULTS:**

Using a quantitative genetic analysis of 400 pedigreed *Alopecurus myosuroides* seed families from nine field‐collected populations, we found strong heritability for resistance to the acetolactate synthase and acetyl CoA carboxylase inhibitors (*h* ^2^ = 0.731 and 0.938, respectively), and evidence for shared additive genetic variance for resistance to these two different herbicide modes of action, *r*_g_ = 0.34 (survival), 0.38 (biomass). We find no evidence for genetic correlations between life‐history traits and herbicide resistance, indicating that resistance to these two modes of action is not associated with large fitness costs in blackgrass. We do, however, demonstrate that phenotypic variation in plant flowering characteristics is heritable, *h* ^2^ = 0.213 (flower height), 0.529 (flower head number), 0.449 (time to flowering) and 0.372 (time to seed shed), demonstrating the potential for adaptation to other nonchemical management practices (e.g. mowing of flowering heads) now being adopted for blackgrass control.

**CONCLUSION:**

These results highlight that quantitative genetics can provide important insight into the inheritance and genetic architecture of NTSR, and can be used alongside emerging molecular techniques to better understand the evolutionary and fitness landscape of herbicide resistance. © 2022 The Authors. *Pest Management Science* published by John Wiley & Sons Ltd on behalf of Society of Chemical Industry.

## Graphical abstract

Quantitative genetics reveals the heritability of flowering traits and shared additive‐genetic variance for resistance to the acetolactate synthase and acetyl CoA carboxylase inhibitors, but no evidence for large fitness costs associated with these resistances.

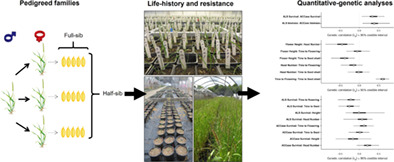

## 1 INTRODUCTION

Reduction in crop yields due to competition from weeds remains one of the greatest constraints to maximising yields in global crop production.^1^ While chemical weed control can be very effective, over‐reliance on herbicides has resulted in a global epidemic of herbicide resistance, with 266 herbicide‐resistant species now reported worldwide.^2^ Throughout Northern and Western Europe, *Alopecurus myosuroides* (blackgrass) is considered to be the most important and costly weed species affecting cereal cropping,^3^, ^4^ with resistance to seven herbicide modes of action (MOAs) reported.^2^ In the UK alone, blackgrass causes an estimated annual economic loss of £0.4 billion due to wheat yield losses of 0.8 million tonnes.^5^ Whilst our understanding of the genetic and evolutionary basis of herbicide resistance is improving,^6^, ^7^, ^8^ there are still many unanswered questions.

Open questions include; does resistance to herbicides with different mechanisms of action (MOAs) share the same genetic basis? And, does the evolution of resistance traits result in other life‐history trade‐offs? Herbicide resistance mechanisms are divided into two main groups. Target‐site resistance (TSR) involves either significantly increased expression of the herbicidal target, or alteration of the structure of the target molecule to reduce herbicide‐target binding. By their nature, such mechanisms only convey resistance to those herbicides which share a biological target. In contrast, changes in the rates of herbicide absorption, translocation or metabolism, termed non‐target‐site resistance (NTSR), can convey broader and more unpredictable cross‐resistance.^9^, ^10^, ^11^, ^12^ The cytochrome P450 monooxygenase (P450s),^13^, ^14^ glutathione S‐transferase (GST)^15^ and ATP‐binding cassettes (ABC) transporter superfamilies^16^ have all been implicated in NTSR to multiple different MOAs across a broad range of species.^8^ In addition, links with broader plant stress responses and reactive oxygen species (ROS) scavenging have been suggested to play a role in minimising herbicide damage and recovery from herbicide injury,^17^, ^18^ contributing to the NTSR mechanism. As such, the underlying genetic basis for NTSR remains poorly understood, but likely involves multiple genes and pathways. Understanding variation in the genetic architecture of NTSR within and among weed populations has been highlighted as a priority for study,^19^ but has remained hampered by the scarcity of genetic resources for these non‐model species.

Typically, there has been a theoretical assumption that evolved resistance mechanisms should result in fitness costs.^20^, ^21^ It has been hypothesised that TSR mutations, which alter the structure of key plant enzymes, might also reduce substrate binding affinity and catalytic capacity, thereby reducing enzyme efficiency,^22^ although evidence of this in practice has been scarce. Overproduction of herbicide targets or molecules involved in herbicide detoxification could also require increased allocation of plant resources, diverting resources away from growth or reproduction. As such, many studies have predicted that TSR or NTSR could result in reduced fitness. Although there are cases in support of fitness costs,^22^, ^23^, ^24^, ^25^, ^26^, ^27^ the prevailing evidence now is that such costs are not universal.^28^, ^29^, ^30^, ^31^, ^32^, ^33^ In some cases, co‐evolution of other plant traits might act to directly compensate for the detrimental fitness effects of resistance alleles,^34^ while in other instances cultural practices, including crop choice and competitive cultivars, used alongside herbicide selection might concomitantly select for greater ‘weediness’, potentially masking small fitness penalties.^35^, ^36^ The possibility that fitness costs (if present) might be small, and the potential for such multitrait co‐evolution highlights the need for careful experimental design, appropriate statistical power and assessment of multiple fitness‐related traits. Much has been written about methodological difficulties and limitations in studies to detect costs associated with herbicide resistance, highlighting low statistical power and issues around uncontrolled genetic backgrounds. Where resistance is polygenic, as suspected for many NTSR traits, gaining meaningful estimates of fitness can be even more problematic due to the relative effects of multiple, generally unknown, alleles. This has led some to call for alternative approaches to measure such costs.^25^, ^37^, ^38^

Quantitative genetics has been highlighted as an approach which might bridge these two areas of research, allowing simultaneous investigation of the additive‐genetic basis and trade‐offs associated with herbicide resistance traits.^29^, ^39^, ^40^ Through analysis of genetic lines with known pedigrees, the additive genetic variance and co‐variance underpinning a set of plant traits can be estimated. Using this approach, estimates of the heritability of herbicide resistance phenotypes can be ascertained,^41^ while genetic correlations can help to reveal the extent of the shared ‘genetic architecture’ underpinning resistance to different herbicide MOAs, and life‐history trade‐offs. As an additional benefit, applying quantitative genetic analyses to field‐evolved weed populations can help to provide evolutionary inference on other aspects of plant life history which might be under selection. For example, a quantitative genetic study of multiple populations of *Amaranthus palmeri* identified several weedy traits under selection in the field.^42^ With the increased adoption of non‐chemical methods and integrated weed management (IWM) for weed control, such understanding of the heritability and potential response to selection for a range of plant traits will be important for predicting future adaptation to weed management.

Here we use a classical quantitative genetics approach to investigate the additive genetic variance and co‐variance underpinning herbicide resistance in the weed *Alopecurus myosuroides*. TSR to the acetolactate synthase (ALS) and acetyl CoA carboxylase (ACCase) inhibiting herbicides has been extensively reported in this species, with mutations particularly frequent at positions 197 (ALS) and 1781 (ACCase).^43^ Notably, NTSR to these two MOAs is also widespread amongst *A. myosuroides* populations, co‐occurring with TSR and potentially causing broader and more unpredictable patterns of resistance.^4^ In the current study, 400 pedigreed seed families from nine field‐collected populations were established, encompassing both TSR and NTSR mechanisms, and used to examine the inheritance and genetic architecture of resistance. We test the hypothesis that there is shared genetic architecture for resistance to the ALS‐ and ACCase‐inhibiting herbicides, indicative of some degree of generalist herbicide cross‐resistance in this species. Through assessment of genetic correlations, we also test the hypothesis that inheritance of the genetic architecture for resistance to either MOA will result in some measurable fitness cost in plant morphology or life history. Finally, investigation of the heritability and additive‐genetic variance and co‐variance amongst multiple plant traits allows us to highlight the capacity for further plant adaptation and trait (co‐)evolution in response to weed management. Comparison of phenotypic trait divergence (*Q*_ST_) with among‐population molecular differentiation (*F*_ST_) allows us to test the hypothesis that such divergent selection is already underway amongst UK *A. myosuroides* populations.

## 2 MATERIALS AND METHODS

### 2.1 Collection of the field populations

**Figure 1:**
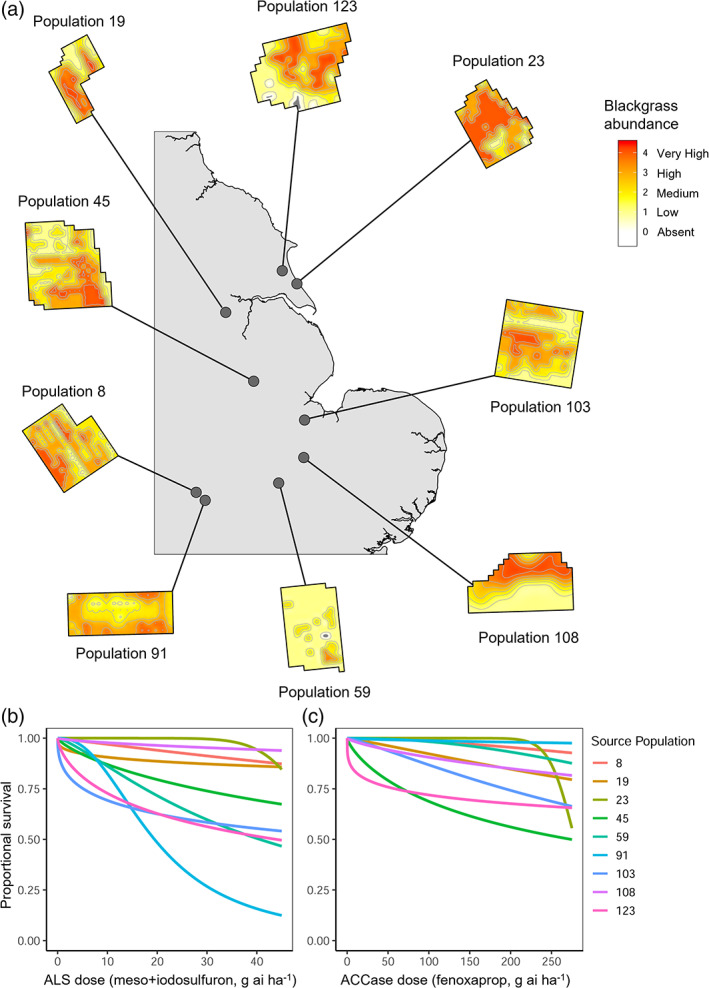
Nine field populations of blackgrass (*Alopecurus myosuroides*) used to generate the pedigreed families. (a) Geographical location of each field, with field maps coloured according to the severity of blackgrass infestation (measured on an ordinal scale from 0 = absent to 4 = very high, see Queenborough *et al*.^74^). Dose–response relationships are also shown for each population after exposure to (b) the ALS inhibitor meso+iodosulfuron, or (c) the ACCase inhibitor fenoxaprop. Proportional survival represents the ratio of the number of plants surviving herbicide *versus* the total number of plants sprayed. For further details of the experimental methodology see Comont *et al*.^43^

Blackgrass in a predominantly autumn‐germinating grassweed infesting cereal crops throughout North‐Western Europe. As an obligate outcrossing species with high fecundity, resistance mutations can spread rapidly and facilitate large population sizes. Nine blackgrass‐infested agricultural fields were visited in the summer of 2014 as part of a wider survey of herbicide resistance.^4^ These nine sites were widely distributed across the main blackgrass areas in England and represent a variety of infestation and herbicide resistance levels (Fig. 1). Ten sampling locations were identified within each field using a stratified random approach, and five mature blackgrass seed heads were collected at each location. In all cases, seed heads were collected from different individual blackgrass plants, with 50 seed heads collected from each field. Seed heads were air‐dried at room temperature for 1 week before carefully removing the ripened, filled seeds.

### 2.2 Creating pedigreed seed families

The collected seeds were used to generate pedigreed seed families with a paternal half‐sibling crossing design. Seeds from each individual field‐collected seed head were germinated in Petri dishes containing two Whatman No. 1 filter papers soaked in 0.02 mol L^−1^ KNO_3_. Petri dishes were incubated for 7 days in a Sanyo MLR‐350 growth cabinet with a 17/11 °C temperature cycle and a 14:10 h light:dark cycle. Individual germinated seedlings from each seed head were transplanted into 15‐cm (1.55‐L) plastic plant pots containing compost. Pots were kept in a 16/14 °C glasshouse between September and November. During December, supplementary heating was turned off, allowing the plants to vernalise at approximately ambient winter temperatures. After vernalisation, the temperature was increased to 20/15 °C with a 14:10 h light:dark day length.

Once plants had established sufficient biomass, one‐quarter of the vernalised plants from each population were randomly chosen to be pollen donors (paternal plants), while the remaining three‐quarters of plants were designated for seed collection (maternal plants). Paternal plants were split into three tillers (genetic clones). Each paternal tiller was then randomly paired with an uncloned (maternal) individual from the same population. At the onset of flowering, paired plants were bagged together using plastic pollination bags, allowing cross‐pollination of seed heads within each pair, but preventing cross‐pollination between different pairs of parental plants. Once seed heads were mature, seeds were collected from each maternal plant. As *A. myosuroides* is an obligate outcrossing species, all seeds from the maternal plant were considered to be the result of pollination from the paired paternal plant. All seeds from a single maternal plant are therefore considered full‐sibling families, while seeds collected from different maternal plants sharing a pollen donor are half‐sibling families. Collected seed families therefore represent a classic full‐sibling, half‐sibling design. Between 30 and 52 seed families were successfully produced in this way for each of the nine source populations, with 400 pedigreed seed families produced in total.

### 2.3 Herbicide resistance screening

To assess herbicide resistance in each seed family, seeds were germinated in Petri dishes as previously described, with individual germinated seedlings transplanted into 7‐cm (0.23‐L) plastic plant pots. Pots were filled with a Kettering loam soil containing 2 kg m^−2^ osmocote fertiliser. A total of 14,000 plants (35 per seed family) were grown in this way to the three‐leaf stage before spraying with herbicide using a fixed‐track sprayer. The spray nozzle (Teejet, 110015VK) was mounted 50 cm above the plants, with boom speed set at 0.33 m s^−1^ applying herbicide at approximately 200 L ha^−1^. Plants from each family were sprayed with either the commercial ALS inhibiting herbicide ‘Atlantis’ (containing a mixture of the sulfonylurea active ingredients mesosulfuron‐methyl + iodosulfuron, hereafter referred to as meso+iodosulfuron) or the ACCase inhibitor ‘Foxtrot’ (containing the active ingredient fenoxaprop‐*P*‐ethyl, hereafter referred to as fenoxaprop). Seven replicate plants (*n* = 7) were sprayed at either a ‘low’ or ‘high’ dose of each herbicide. For the pre‐formulated mixture of meso+iodosulfuron, the low dose was 14.4 g a.i. ha^−1^ (combined weight of both active ingredients) representing 1× the UK field rate, while the high dose was 43.2 g a.i. ha^−1^, 3× the field rate. For fenoxaprop the low dose was 69 g a.i. ha^−1^, representing 1× the field rate, while the high dose was 207 g a.i. ha^−1^, 3× the field rate. A further seven plants per family were grown as unsprayed controls. Four weeks after spraying, plants were scored as alive or dead, and all aboveground biomass was harvested and weighed following oven drying at 80 °C for 48 h.

Due to constraints on space, all 400 seed families could not be assayed in a single experimental run. Instead, the experiment was performed three times, each time testing a third of the seed families. Approximately equal numbers of families from each of the nine source populations were assessed in each experimental run, and half‐sibling families were not split between runs. Within each run, the plants were spread over three adjacent glasshouse rooms, each set to maintain approximate day/night temperatures of 16/11 °C for the duration of the experiment. Plant position within each glasshouse was determined using an incomplete randomised blocking (alpha) design. This was used to ensure that replicate plants of each family were randomly, but approximately evenly, distributed across all the available glasshouse space, avoiding any undesired grouping of replicates that can occur with a fully random design.

### 2.4 Life‐history assays

A further seven plants of each seed family (2,800 plants in total) were pre‐germinated and sown individually into 15‐cm (1.55‐L) plastic pots containing Kettering loam soil. These plants were kept outdoors under ambient environmental conditions in a netted enclosure to exclude vertebrate pests and birds. Plants were placed in the enclosure from the emergence of their first aboveground leaf in autumn through to reproductive maturity the following summer (02 November 2016 to 03 July 2017). Replicates from each family were organised in randomised blocks along the length of this area. Daily supplementary watering was provided by overhead sprinklers from late spring to prevent excessive soil drying.

Plants were assessed non‐destructively on 10 occasions during establishment and vegetative growth over winter/spring (15 December 2016 to 15 March 2017). At each interval, the number of tillers and length of the longest tiller were recorded for each plant. Flowering time was recorded as the time at which the first flower spike became visible after emerging from the surrounding leaf‐sheath. The commencement of seed shedding was also recorded for each plant. These dates were converted into thermal growing degree‐days using meteorological data recorded daily on‐site (Harpenden, UK). A base temperature of 1 °C was used.^44^ On 03 July 2017, once all plants had flowered, the total number of seed‐heads produced and the length of the longest flowering tiller were recorded for each plant.

Mature seeds were collected from all plants, and seeds from the seven replicate plants per family were combined to create a single, family‐level seed bulk. Three replicates of 50 seeds were counted for each seed bulk and weighed to determine the 50‐seed weight. Not all *A. myosuroides* seeds contain a viable caryopsis.^45^ To calculate the proportion of such non‐viable, unfilled seeds, a seed squash test was used. Four replicate sets of 25 dry seeds from each seed bulk were distributed evenly over a sheet of card, stuck down using clear adhesive tape and rolled flat using a heavy weight. Using this method, the filled caryopsis of viable seeds is squashed out, making these easily distinguishable from unfilled, non‐viable seeds. Using this method, the proportion of viable, filled seeds was calculated for each family‐level seed bulk. This value was used to correct the proportion of seed that had the capacity to germinate in the calculation of germinability.

To determine germinability, three technical replicates of 50 or more seeds were plated on 0.8% water agar Petri dishes and incubated for 2 weeks at 16/10 °C with a 14:10 h light:dark cycle in a Sanyo MLR‐350 growth cabinet. The Petri dishes were stacked so that the technical replicates were distributed across different stacks, and the stacks were rotated every other day within the growth cabinet to ensure equal exposure to light. At the end of this time, seeds were counted as germinated if the radicle or shoot had visibly emerged through the seed coat. Binocular dissecting microscopes were used to facilitate observation of very early emergence. Germination frequency was calculated as the number of seeds with a visibly protruding radicle or shoot divided by the proportion of the seed that had the capacity to germinate (after correction according to the seed‐filling test results).

### 2.5 Assessment of among‐population variability

To assess the magnitude of among‐population variance in life‐history and resistance traits, an initial set of univariate Bayesian mixed‐effects models were fitted. In each case, ‘source population’ (the ID of the nine field‐collected populations that the lines were derived from) was included as a fixed effect, while ‘seed family ID’ was included as a random term. Other sources of experimental variance were included as fixed effects, with models for herbicide resistance traits including fixed‐effect terms for experimental run, glasshouse compartment and the glasshouse × run interaction, while models for life‐history characteristics included a fixed‐effect term for the experimental block. For binary traits such as herbicide survival where the residual variance is inestimable, the value of *V*_R_ was fixed at 1. All models were fitted in R version 3.4.2 using the package MCMCglmm.^46^ In all cases, models were fitted using parameter expanded priors and run for 5 million iterations with the burn‐in and thinning varied to maximise chain mixing within each model. The posterior means and 95% highest posterior density (HPD) intervals were calculated for each source population, and significant differences were ascribed to populations with nonoverlapping HPD intervals.

### 2.6 Quantitative genetic analyses

Analysis of quantitative genetic parameters was performed using a Bayesian animal‐modelling approach.^47^ Preliminary assessment of herbicide phenotyping data and models showed that responses to both high and low doses of the same herbicide were very similar, with a very high genetic correlation (Fig. S1). Therefore, data from both doses was combined into single ‘biomass’ and ‘survival’ response variables for each herbicide in subsequent models. After trialling a range of models with different life‐history traits and/or resistance measures included, a set of four multivariate mixed‐models was constructed. The response variables included in the four models were (1) bivariate measures of resistance (fenoxaprop survival, meso+iodosulfuron survoival), (2) measures of biomass after herbicide screening (control biomass, fenoxaprop biomass, meso+iodosulfuron biomass), (3) repeated measures assessment of vegetative development (tiller number, tiller length) and (4) flowering characteristics (flower head number, flower head height, thermal time to flowering, thermal time to seed shedding). Multivariate models containing a greater range of the measured traits were trialled, but not evaluated due to poor chain‐mixing and posterior distribution estimates in some cases.

As previously, potential sources of experimental variance were included as fixed effects, such that models (1) and (2) included fixed‐effects terms for the herbicide dose, experimental run, glasshouse compartment and the glasshouse × run interaction, while models (3) and (4) included a fixed‐effect term for the experimental block. All models included random‐effect terms for ‘animal’ (additive genetic variance), ‘mother’ (maternal variance) and ‘source population’. Model (3) included an additional random effect term ‘plant ID’ to account for the repeated‐measures nature of the data. As above, the value of *V*_R_ (residual variance) was fixed at 1 for binary traits, while residual covariance was fixed at zero where the response variables were measured on different individual plants. Models were fitted using parameter expanded priors and run for 5 million iterations with the burn‐in and thinning varied to maximise chain mixing within each model. Posterior distributions and chain mixing were assessed for all models before interpretation.

To evaluate the potential for genetic correlation between herbicide resistance and plant life history, a further set of bivariate animal models were fitted. Plant survival after herbicide application was used as the estimate of resistance. For each of the measured plant life‐history characteristics, a separate bivariate animal model was constructed containing a single life‐history trait and herbicide resistance to either meso+iodosulfuron or fenoxaprop. As above, sources of experimental variability were included as fixed effects, with random terms included to estimate the additive genetic variance (*V*_A_), maternal variance (*V*_M_) and among‐population variance (*V*_B_).

The additive genetic variance (*V*_A_) and total phenotypic variance (*V*_P_) were extracted from the fitted models following the approach of Wilson *et al*.,^47^ as:

(1) $$V_{P}=V_{A}+V_{M}+V_{R}$$

where *V*_A_ is the additive genetic variance for a trait, *V*_M_ is the maternal variance and *V*_R_ is the residual variance. Among‐population variance (*V*_B_) was estimated in the model but not included in the calculation of the total phenotypic variance. For binomial traits the *V*_R_ is inestimable and was fixed as 1. Narrow‐sense heritability (*h* ^2^) was calculated by dividing the additive genetic variance (*V*_A_) by the total phenotypic variance (*V*_P_), as:

(2) $$h^{2}=\frac{V_{A}}{V_{P}}$$

The genetic correlation between two traits (*r*_G_) was calculated from the additive genetic variances and co‐variances as specified in Wilson *et al*.^47^:

(3) $$r_{G}=\frac{{\mathrm{COV}}_{A_{1,2}}}{\sqrt{V_{A_{1}}\times V_{A_{2}}}}$$

where ${\mathrm{COV}}_{A_{1,2}}$ represents the additive genetic covariance between traits 1 and 2, while $V_{A_{1}}$ and $V_{A_{2}}$ are the additive genetic variances of traits 1 and 2, respectively. The among‐population differentiation for each phenotypic trait (*Q*_st_, analogous to *F*_st_ for genetic traits) was calculated using the between‐population (*V*_B_) estimates of variance as given in Wood *et al*.^48^:

(4) $$Q_{\mathrm{st}}=\frac{V_{B}}{V_{B}+\left(2\times V_{A}\right)}$$

where *V*_B_ is the between‐population variance and *V*_A_ is the additive genetic variance for a particular trait.  All of the estimates of heritability (*h*_2_), genetic correlation (*r*_G_) and among‐population differentiation (*Q*_st_) were calculated on the latent scale using the variance–covariance matrix directly from the fitted MCMCglmm models. For calculation of evolvability (*I*_A_), first the latent additive genetic variances (*V*_A_) were transformed back to the observed data scale using the ‘QGmvparams’ function in the ‘QGglmm’ package.^49^ This step is necessary to account for variance introduced by the family and link functions of the GLMM, and allows mean standardisation of the variances using the observed phenotypic data. The mean‐standardised evolvability was then calculated as given in Hansen *et al*.^50^:

(5) $$I_{A}=\frac{V_{A_{\mathrm{obs}}}}{{\underline{x}}^{2}}$$

where *V*_Aobs_ represents the additive genetic variances after transformation back to the observed data scale, while the denominator ${\underline{x}}^{2}$ is the squared mean of the phenotypic trait.

## 3 RESULTS

### 3.1 Variability in resistance and life‐history characteristics

**Figure 2:**
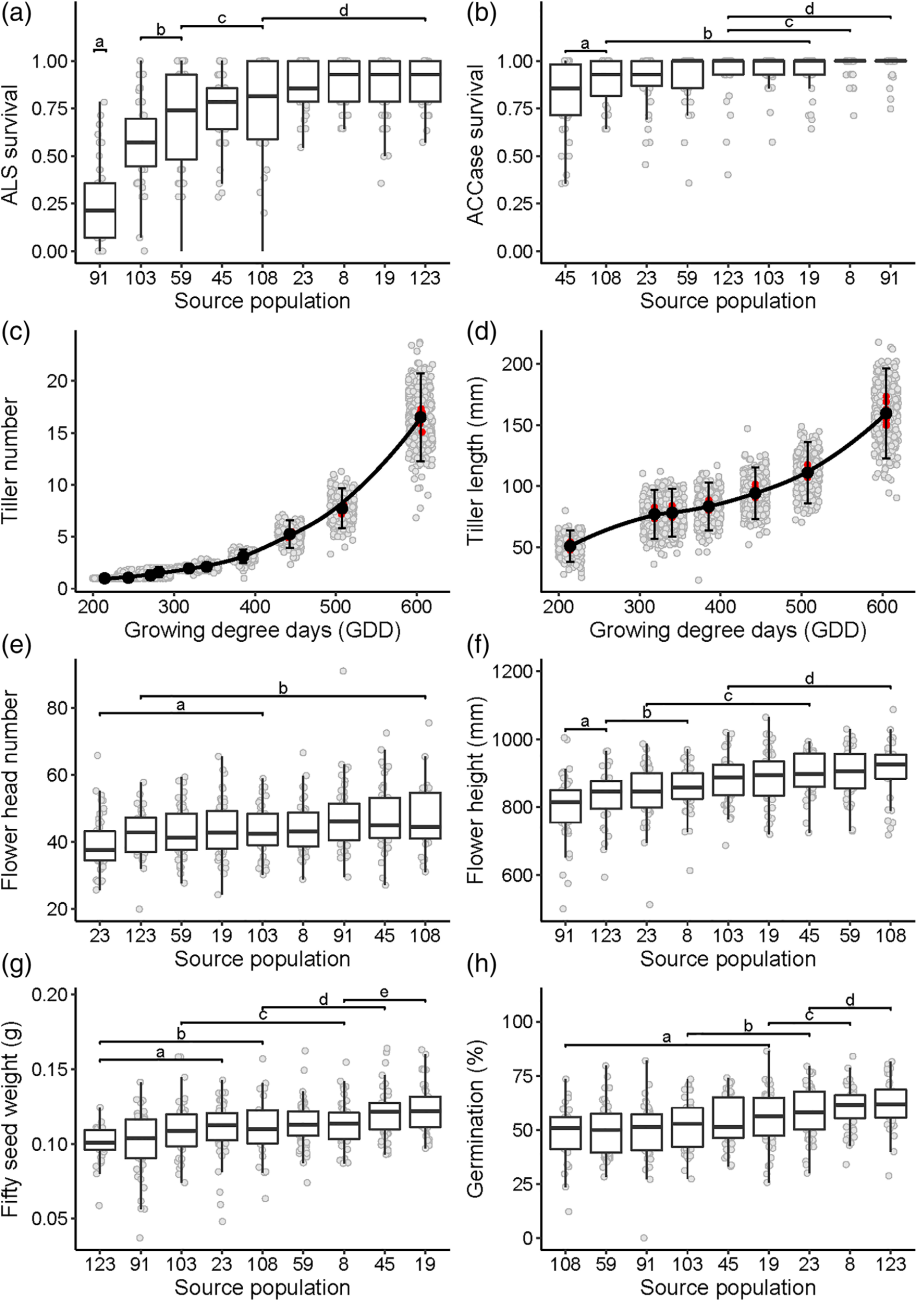
Variation in herbicide resistance, calculated as proportional survival of herbicide applications (a, b), vegetative development (c, d), flower production (e, f), and seed production (g, h) amongst the seed families. Boxplots show the median and interquartile ranges, while points show the mean value for each of the 400 seed families. Letters denote significant differences, assessed as non‐overlapping 95% HPD intervals. For (c) and (d), Light grey points show the means for each seed family, while red points show the mean of all the families from each source population.

Significant effects of source population were observed across all stages of plant life history, although differences were generally small in comparison to the variability amongst seed families within source populations (Fig. 2, and see Tables S1 and S2). Significant variation in herbicide resistance was observed, both within and between seed families from each source population (Fig. 2(a),(b)). Greater variation in resistance to the ALS inhibitor (meso+iodosulfuron) was found, with families ranging from 0% to 100% survival, even amongst seed families from the same source population. In contrast, ACCase (fenoxaprop) resistance was more frequent across the seed families, although significant variation was still observed amongst seed families from different source populations (Fig. 2).

Analysis of vegetative growth data found that increases in tiller number followed an approximately exponential relationship with thermal time, while tiller length followed a polynomial relationship (Fig. 2(c),(d)). For both traits, there was substantial variation within and between seed families, although differences at the level of the source population were very small, particularly during the early stages of vegetative development (Fig. 2 and Table S1). Significant differences amongst the nine source populations were observed for reproductive traits, including in flower head number and height (Fig. 2(e),(f)), and in the weight and germinability of produced seeds (Fig. 2(g),(h)).

### 3.2 Heritability and evolvability of resistance and life‐history characteristics

Resistance to both herbicides was strongly heritable, with survival data giving higher heritability estimates than biomass (Table 1). Tiller length during vegetative development had a very weak heritable basis and tiller number showed almost no heritable variation. In contrast, all reproductive traits had significant heritability, with the number of flower heads in particular being strongly heritable at *h^2^ = 0.53* (Table 1). The mean‐standardised estimates of evolvability were uniformly low with the exception of resistance to the herbicide meso+iodosulfuron, measured as plant biomass after herbicide application (Table 1).

**Table 1:** Narrow‐sense heritability estimates (*h* ^2^) and mean‐standardised evolvability (*I*_A_) for herbicide resistance and life‐history characteristics in blackgrass

Trait	*h* ^2^	Lower	Upper	*I*_A_	Lower	Upper
*Herbicide resistance*						
Survival: meso+iodosulfuron	0.731	0.469	0.907	0.007	0.000	0.036
Survival: fenoxaprop	0.938	0.828	0.953	0.001	0.000	0.006
Biomass: meso+iodosulfuron	0.432	0.312	0.531	0.408	0.285	0.519
Biomass: fenoxaprop	0.394	0.121	0.589	0.151	0.042	0.223
*Vegetative development*						
Tiller length	0.054	0.012	0.097	0.010	0.002	0.017
Tiller number	0.000	0.000	0.001	0.000	0.000	0.001
*Flowering*						
Flower height	0.213	0.072	0.305	0.006	0.002	0.009
Flower head number	0.529	0.243	0.688	0.034	0.015	0.050
Time to flowering	0.449	0.289	0.546	0.002	0.002	0.003
Time to seed shed	0.372	0.235	0.473	0.001	0.001	0.002

Herbicide resistance traits are the survival or dry weight of plants taken 4 weeks after spraying with either the ALS inhibitor meso+iodosulfuron or the ACCase inhibitor fenoxaprop. Posterior modes are given, along with the lower and upper 95% credible intervals.

### 3.3 Genetic correlation between resistance to different herbicide MOAs and life‐history traits

**Figure 3:**
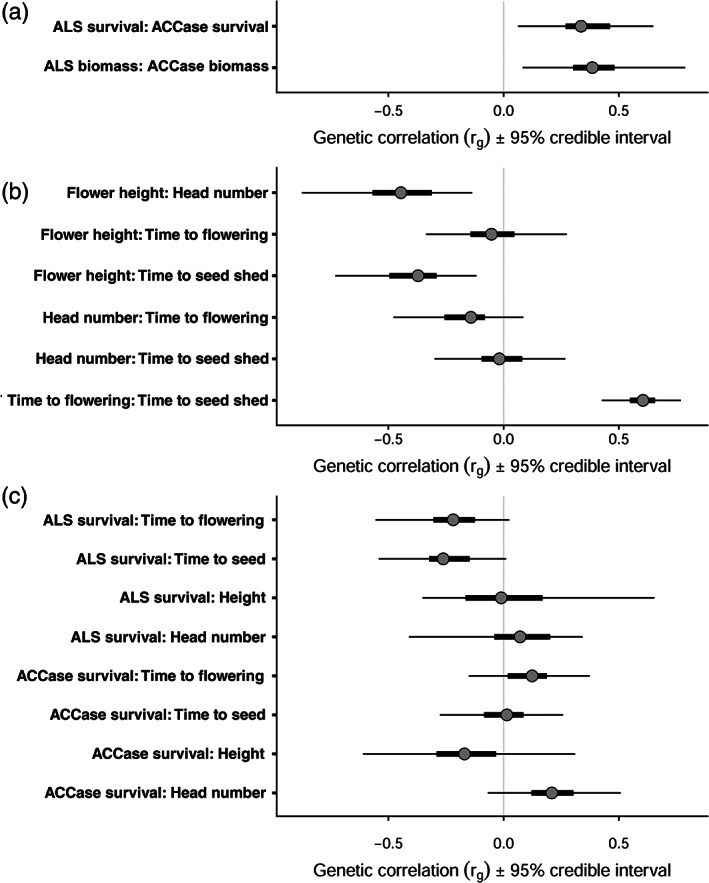
Genetic correlations (*r*_g_) amongst blackgrass herbicide resistance and life‐history traits (a) between resistance to the herbicides meso+iodosulfuron and fenoxaprop, (b) between flowering traits, and (c) between herbicide resistance and flowering traits. Posterior modes are shown, with thick and thinner bars showing the 50% and 95% credible intervals, respectively.

A significant, positive genetic correlation was observed between resistance to meso+iodosulfuron and fenoxaprop, using both survival and biomass measurements (Fig. 3(a)). This clearly demonstrates a shared genetic architecture for resistance to these two herbicide MOAs. There were, however, no significant genetic correlations between herbicide resistance and flowering traits, although HPD intervals for negative correlations between resistance to meso+iodosulfuron and phenological timings for flowering and seed shed were almost significant (Fig. 3(c)). There is no evidence therefore that the genetic architecture underpinning resistance to these two herbicides is associated with any trade‐off in reproductive traits, which would have been strongly indicative of fitness costs.

Some genetic correlations were observed amongst the life‐history data, however. The strongest genetic correlation was between the thermal time to first flower emergence and first seed shed. The correlation was positive, i.e. plants which flower earlier also set seed earlier. Flower height had negative genetic correlations with both the total number of flower heads and time to seed shed (Fig. 3(b)). This suggests a genetic trade‐off whereby plants which produce longer flowering tillers have fewer flower heads and shed seeds later.

### 3.4 Among‐population divergence (*Q*_st_)

**Figure 4:**
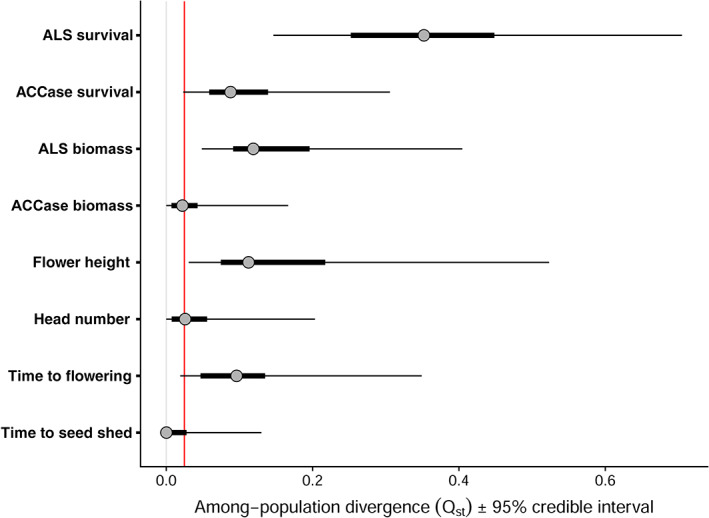
Among‐population divergence in phenotypic traits (*Q*_st_) calculated according to Eqn 4 following MCMC glmm analysis. Points represent the posterior modes, with thick and thinner horizontal bars showing the 50% and 95% credible intervals, respectively. The vertical red line shows the mean pairwise *F*_st_ from a broader assessment of 43 UK blackgrass populations collected alongside the nine source populations in this study.^51^

Resistance to fenoxaprop was high amongst seed families from all nine of the field‐collected source populations, whereas resistance to meso+iodosulfuron showed greater variability (Fig. 1(a),(b)). Concomitantly, *Q*_st_ values for resistance to meso+iodosulfuron were higher than for resistance to fenoxaprop, suggesting that there are greater differences among the nine field‐collected source populations in their additive‐genetic variance for meso+iodosulfuron resistance (Fig. 4). Although *F*_st_ was not calculated in this study, the source populations here form part of a larger study of blackgrass population structure (including *F*_st_ estimation) in the UK,^51^ facilitating some estimated *F*_st_ to *Q*_st_ comparison. Amongst the four flowering traits, the height of the flowering tiller had a moderate *Q*_st_ value, with an HPD interval above the mean pairwise *F*_st_ (Fig. 4), while time to flowering also neared significance. This may signify some selection acting on flowering strategy amongst these populations.

## 4 DISCUSSION

Understanding the genetic basis underlying plant resistance to herbicides has long been a topic of study for weed biologists. However, despite advances in understanding the biochemical basis of enhanced metabolism‐based herbicide detoxification,^52^, ^53^ little is currently known about the genetic variation and co‐variation underlying NTSR mechanisms^7^, ^8^, ^19^, ^54^, ^55^ or the consequences of resistance on life‐history trade‐offs.

One particular question surrounds the heritability and extent to which NTSR to different herbicide MOAs shares the same underlying genetic basis (e.g. mutations, alleles, quantitative trait loci). In grass weeds, the same protein superfamilies, including the GSTs^15^ and P450s,^56^ are repeatedly implicated in resistance to a range of MOAs, and populations with NTSR to multiple MOAs are widespread.^57^, ^58^, ^59^, ^60^ However, the extent to which such multiple resistance shares the same genetic architecture remains incompletely resolved. As expected, the results of the current study highlight extremely high heritability estimates for resistance to both the ACCase inhibitor fenoxaprop and the ALS inhibitor meso+iodosulfuron. As resistance is in part provided by single‐locus dominant TSR mutations, a high heritability is unsurprising, although the controlled experimental conditions may facilitate a higher heritability estimate here than might be observed in the field. Interestingly, however, a significant positive genetic correlation was also observed between resistance to these separate herbicide groups, *r*_g_ = 0.34 (survival) and *r*_g_ = 0.38 (biomass). Given the MOA‐specific resistance (specialist resistance) provided by TSR mutations,^61^ it is most likely that this represents an element of shared genetic architecture for NTSR to these two MOAs. As MOA‐specific TSR is also present amongst these populations, the true additive genetic correlation between NTSR to the ALS and ACCase herbicides tested might in fact be even higher than shown here. Whilst this does not mean that NTSR to these two herbicides is identical, it supports evidence that the non‐target‐site mechanism of resistance to these different herbicide MOAs may involve some of the same underlying alleles.^43^, ^58^, ^60^

From an evolutionary perspective, these results may help to explain why resistance to the ALS inhibitors has evolved so rapidly in UK populations of blackgrass. Using experimental evolution approaches, pre‐selection with one herbicide MOA has been shown to result in genotypes which responded more rapidly to selection by another MOA in the unicellular algae *Chlamydomonas reinhardtii*.^62^ In the UK, use of ACCase inhibitors for control of *A. myosuroides* pre‐dates ALS inhibitor use and so many populations exposed to ALS herbicides will have had prior exposure to ACCase inhibitors. We hypothesise, based on the genetic correlations found here, that widespread selection with ACCase inhibitors will have pre‐selected for the rapid evolution of ALS resistance. While GSTs have been implicated in the metabolism of ACCase herbicides in *A. myosuroides*,^15^ cytochrome P450s have also been linked with resistance in this species.^63^ Emerging evidence from other species now suggests that overexpression of certain P450 enzymes can convey simultaneous cross‐resistance to multiple herbicide MOAs, including both the ACCase and ALS herbicide groups.^13^, ^64^ Further molecular genetic study is warranted to unpick this genetic correlation and identify its transcriptional and mutational basis. Such results raise additional concerns over the extent to which current NTSR genotypes may also have pre‐selected for resistance to novel, as‐yet unreleased, chemistry.

Evidence for costs and trade‐offs associated with evolution of herbicide resistance remains ambivalent.^25^ Polygenic traits in particular (as many NTSR mechanisms are suspected to be) involve the contribution of multiple, potentially population‐specific, alleles. As such, the extent of resistance, and any associated consequences for plant fitness, needs to be considered in relation to the overall genetic architecture of the plant or population. Classical quantitative genetics approaches provide a novel means to address this issue.^40^ In terms of plant life‐history characteristics, we found that vegetative traits had relatively low heritability, but that reproductive traits were more strongly heritable. In particular, the narrow sense heritability for flower head number was high (*h*_2_ = 0.53) and comparable with that observed in other weed species.^65^ Although a negative relationship between ALS (meso+iodosulfuron) resistance and timings of flowering and seed shed was almost significant, overall we found no evidence of significant genetic correlations between herbicide resistance and any measured life‐history trait. While some previous studies have shown fitness costs for NTSR mechanisms,^23^, ^26^, ^66^, ^67^ the current results concur with growing evidence that such results are not ubiquitous. In *Apera spica‐venti*, NTSR to the ALS herbicides was found to convey no associated fitness cost^32^ and NTSR to the herbicide atrazine cause no reduction in fitness in the species *Amaranthus tuberculatus*.^68^ In *A. myosuroides*, previous investigation has similarly found no direct costs in growth or fecundity associated with NTSR to the ACCase herbicides^33^ or the ALS herbicides.^69^ The benefit of the current quantitative genetics approach over these previous studies is that it incorporates multiple populations with potentially independent origins of resistance whilst also providing control for differences in genetic background,^40^ providing an overview of genetic correlations at a level above that of the individual population. In so doing, we confirm that there are no pronounced fitness costs associated with NTSR to the ACCase and ALS herbicides in the blackgrass populations and under the experimental conditions tested here, although it should be noted that the potential for genotype × environment interactions means that we cannot rule out fitness penalties occurring under other environmental conditions.

Finally, with nonchemical practices increasingly employed for blackgrass control,^70^ it is interesting to speculate if plant adaptation may occur to counter these management techniques. One practice gaining increased adoption for blackgrass control is the use of cutting headers, which remove flowering heads which emerge above the crop.^71^ However, this in turn may provide a selective pressure for shorter flowering tillers, and anecdotal evidence from growers employing these techniques for *A. myosuroides* control has been that populations can rapidly (over 2–3 years) exhibit a shorter‐stemmed flowering morphology. In this study flower height was heritable (*h* ^2^ = 0.21) and the estimate of among‐population divergence (*Q*_st_) was greater than any of the other measured floral traits (*Q*_st_ = 0.11). Comparison of *Q*_st_ with an estimated *F*_st_ ^51^ suggests that these differences may be greater than expected due to drift alone, indicating that this divergence may already represent a response to selection in the field. The potential for evolutionary response to non‐chemical control has previously been shown in *Raphanus raphanistrum* (wild radish), with earlier flowering and reduced flowering height demonstrated through experimental recurrent selection, highlighting the possibility for these adaptive traits to evolve to comparable in‐field selection (e.g. via the use of harvest weed‐seed control).^72^ The heritability of flowering time and the presence of a negative genetic correlation between flower height and time to seed shed suggests that a similar response could be predicted in blackgrass. While these analyses do not provide direct evidence of adaptation to non‐chemical controls, they highlight that the evolutionary potential for such adaptation is present in this species and the value in quantitative genetics approaches for proactively identifying this potential.

## 5 CONCLUSION

In conclusion, we have demonstrated the utility of a classical quantitative genetics approach to provide important insight into the inheritance and genetic landscape of resistance in *A. myosuroides*. The observation of shared additive genetic variance for resistance to two different herbicide MOAs lends weight to concerns that resistance mechanisms with broader, more generalist cross‐resistance are evolving in this species.^19^, ^43^ The lack of significant genetic correlations of resistance with plant life history also supports observations that the evolution of such resistance is not accompanied by consistent reductions in plant fitness.^33^, ^69^ Moreover, we are able to demonstrate the heritable nature of plant traits that may be directionally selected by the adoption of non‐chemical controls, highlighting the potential for evolutionary adaptation to these management practices. Overall, our results highlight the importance of a weed species' genetic architecture and additive‐genetic correlations in shaping response to selection by agronomic management, within and between populations. Although the quantitative genetic framework employed is time and resource heavy, it overcomes some of the criticisms directed at more traditional methods^37^, ^38^ and could be used alongside emerging molecular techniques^73^ to better understand the evolutionary and fitness landscape in herbicide‐resistant species.

## CONFLICT OF INTEREST

All authors declare no conflicts of interest.

## Supporting information


**Appendix S1:** Supporting Information

## Data Availability

The data that support the findings of this study are available from the corresponding author upon reasonable request.
