# Supplementary material for: Dissecting weed adaptation: Fitness and trait correlations in herbicide‐resistant Alopecurus myosuroides
Source: Pest Manag Sci. 2022 May 9;78(7):3039–50. doi: 10.1002/ps.6930 (PMC9324217; doi:10.1002/ps.6930)
Supplement: Supplementary file 1 — Appendix S1: Supporting Information [file PS-78-3039-s001.docx]

**Supporting information**


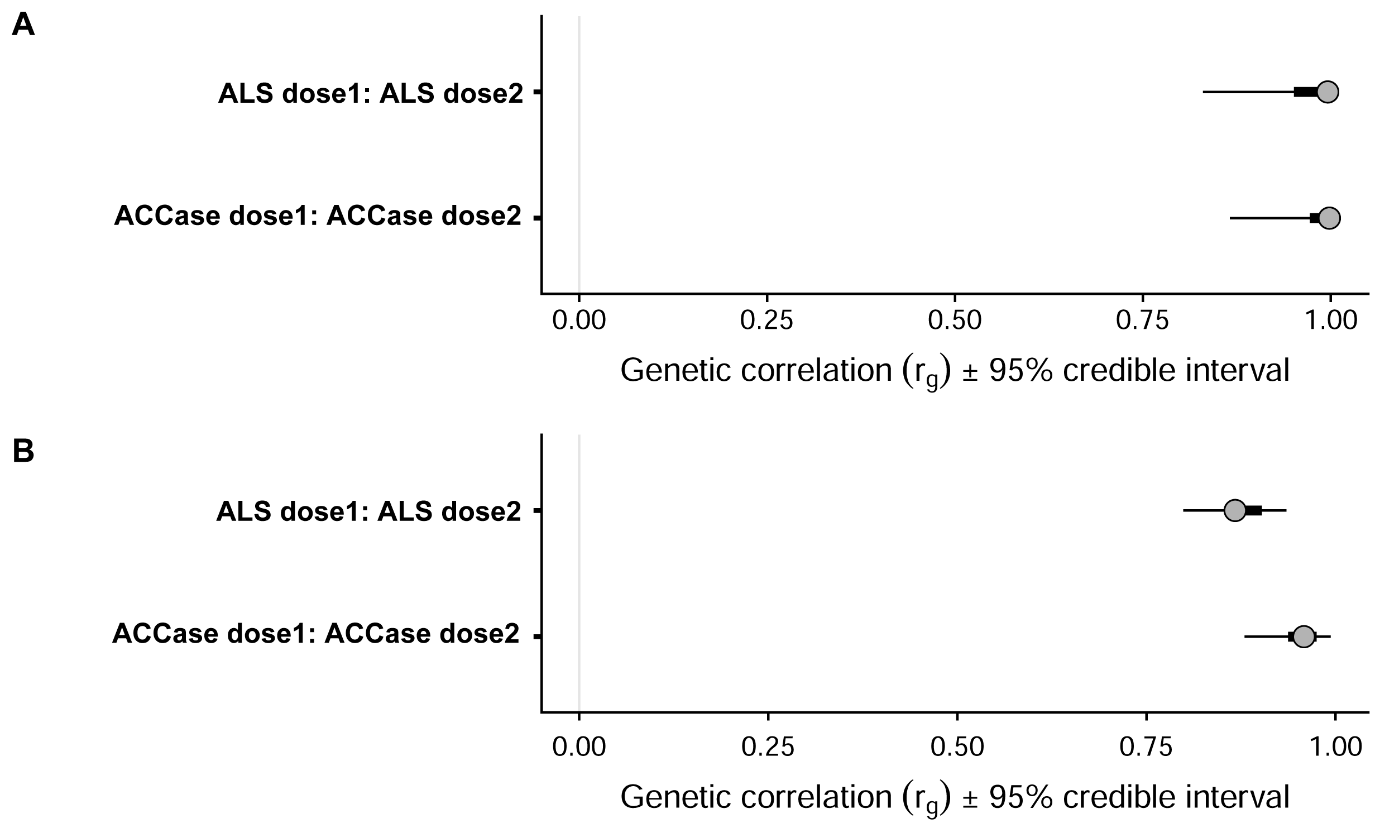


**Figure S1:** Genetic correlations (r_G_) from preliminary models of the herbicide phenotyping data. Responses to each dose of herbicide were treated as separate traits in models of (A) plant survival, and (B) aboveground biomass. Posterior modes are shown, along with the 50% (thick line) and 95% (thinner line) HPD intervals.

**Table S1:** Means and standard deviations (in brackets) of all measured life history characteristics. Values are calculated from all measured seed families derived from each of the nine source populations. The abbreviation ‘GDD’ stands for thermal time in growing degree days above a base temperature of 1°C.

|  | **Source population** | | | | | | | | |
| --- | --- | --- | --- | --- | --- | --- | --- | --- | --- |
| Trait | **8** | **19** | **23** | **45** | **59** | **91** | **103** | **108** | **123** |
| Height (214 GDD) | 49.5  (10.4) | 49.9  (12.4) | 47.5  (12.4) | 53.7  (11.2) | 50.8  (14.7) | 49.1  (13.3) | 50.4  (14.1) | 54.3  (12.6) | 53.9  (13.2) |
| Height (318 GDD) | 74.8  (17.1) | 75.1  (17.9) | 73.1  (19.5) | 80.2  (18.8) | 77.5  (21.2) | 75.6  (21.2) | 77.1  (21.5) | 82.7  (21.3) | 78.5  (21) |
| Height (340 GDD) | 75.7  (16.6) | 76.6  (17.5) | 74.3  (18.8) | 80.9  (18.6) | 78.4  (21) | 77.6  (20.5) | 78  (21) | 84  (20.8) | 79  (20.3) |
| Height (385 GDD) | 80.1  (17.2) | 80.9  (17.6) | 80  (19.3) | 87.1  (18.8) | 84.5  (20.6) | 82.5  (19.8) | 84.4  (21) | 88.4  (21.8) | 83.6  (19) |
| Height (443 GDD) | 91.5  (18.9) | 90.5  (20.2) | 90.6  (21.1) | 99.1  (22) | 95.7  (20.5) | 92.2  (21.6) | 95  (22.8) | 101  (22.4) | 92.2  (20.4) |
| Height (508 GDD) | 108  (22.5) | 108  (22.9) | 107  (25.2) | 117  (24.4) | 113  (24.7) | 107  (25.4) | 113  (26.6) | 117  (28.3) | 110  (24.2) |
| Height (605 GDD) | 151  (31.3) | 155  (35) | 154  (36.3) | 173  (36.9) | 163  (35.7) | 150  (37.2) | 163  (36.4) | 169  (39.4) | 158  (35.3) |
| TillerNo (243 GDD) | 1.04  (0.188) | 1.01  (0.12) | 1.03  (0.179) | 1.05  (0.218) | 1.04  (0.199) | 1.03  (0.167) | 1.1  (0.294) | 1.05  (0.223) | 1.19  (0.391) |
| TillerNo (271 GDD) | 1.29  (0.457) | 1.17  (0.38) | 1.23  (0.424) | 1.35  (0.479) | 1.35  (0.476) | 1.19  (0.392) | 1.36  (0.48) | 1.23  (0.421) | 1.47  (0.5) |
| TillerNo (281 GDD) | 1.6  (0.49) | 1.51  (0.501) | 1.49  (0.501) | 1.68  (0.467) | 1.7  (0.459) | 1.5  (0.501) | 1.68  (0.468) | 1.52  (0.501) | 1.7  (0.461) |
| TillerNo (318 GDD) | 1.97  (0.205) | 1.96  (0.239) | 1.92  (0.299) | 1.97  (0.228) | 1.96  (0.26) | 1.93  (0.269) | 1.95  (0.282) | 1.95  (0.269) | 2.01  (0.367) |
| TillerNo (340 GDD) | 2.13  (0.376) | 2.06  (0.325) | 2.11  (0.429) | 2.17  (0.445) | 2.1  (0.4) | 2.05  (0.332) | 2.14  (0.46) | 2.07  (0.381) | 2.21  (0.467) |
| TillerNo (385 GDD) | 3.15  (0.608) | 3.04  (0.599) | 3.01  (0.659) | 3.21  (0.615) | 3.15  (0.698) | 2.97  (0.593) | 3.12  (0.729) | 3.11  (0.657) | 3.18  (0.745) |
| TillerNo (443 GDD) | 5.31  (1.21) | 5.14  (1.18) | 5.25  (1.35) | 5.45  (1.23) | 5.48  (1.33) | 4.9  (1.26) | 5.19  (1.44) | 5.16  (1.25) | 5.45  (1.47) |
| TillerNo (508 GDD) | 7.77  (1.71) | 7.51  (1.66) | 7.78  (2.07) | 8.07  (1.72) | 7.99  (1.91) | 7.19  (1.76) | 7.8  (2.18) | 7.52  (1.84) | 7.98  (2.26) |
| TillerNo (605 GDD) | 16.8  (3.83) | 15.9  (3.64) | 16.6  (4.27) | 17.3  (3.92) | 17.1  (4.35) | 15.1  (3.95) | 16.7  (4.68) | 15.9  (4.14) | 17  (4.95) |
| Flowering time (GDD) | 995  (69.9) | 942  (72.7) | 980  (67.3) | 928  (68.8) | 969  (61.6) | 972  (76.7) | 990  (73.9) | 968  (91.2) | 978  (69) |
| Seed shed time (GDD) | 1545  (90.2) | 1526  (98.3) | 1533  (85.7) | 1516  (101) | 1528  (88.5) | 1561  (109) | 1553  (97.5) | 1552  (97.2) | 1527  (101) |
| Head number | 44.2  (14.4) | 43.8  (15.4) | 39.2  (12.8) | 47.4  (16.2) | 42.9  (13.5) | 47.4  (16.4) | 43.7  (14.4) | 47.5  (16.7) | 42.5  (13.2) |
| Height (mm) | 859  (156) | 887  (145) | 846  (146) | 898  (140) | 904  (145) | 808  (163) | 881  (142) | 915  (149) | 832  (154) |
| Seed filling (%) | 82.9  (12.6) | 90.2  (7.71) | 87.9  (12.9) | 86  (10.8) | 88.5  (11.2) | 76.7  (23.8) | 86.3  (11.5) | 86.1  (11.8) | 85.5  (13.1) |
| Fifty seed weight (g) | 0.114  (0.0155) | 0.123  (0.0169) | 0.11  (0.0186) | 0.121  (0.0171) | 0.113  (0.0156) | 0.101  (0.0218) | 0.11  (0.0205) | 0.112  (0.0214) | 0.1  (0.0128) |
| Germination (%) | 60.2  (12) | 55.3  (13.9) | 57.9  (13.5) | 53.7  (13.5) | 50  (12.8) | 49.2  (15.7) | 52.2  (13.1) | 48.1  (14.9) | 61.7  (13.3) |

**Table S2:** Means and standard deviations (in brackets) of all measured herbicide phenotyping characteristics. Values are calculated from all measured seed families derived from each of the nine source populations. ‘meso+iodosulfuron ’ is a commercial formulation of the acetolactate synthase (ALS) inhibiting herbicide actives mesosulfuron-methyl and iodosulfuron, while ‘fenoxaprop’ is an acetyl-CoA carboxylase (ACCase) inhibitor. Doses shown are in g ha^-1^ of the active ingredients.

|  | **Source population** | | | | | | | | |
| --- | --- | --- | --- | --- | --- | --- | --- | --- | --- |
| Trait | **8** | **19** | **23** | **45** | **59** | **91** | **103** | **108** | **123** |
| Survival: meso+iodosulfuron (14.4 g ha^-1^) | 0.936  (0.245) | 0.933  (0.25) | 0.973  (0.163) | 0.846  (0.362) | 0.735  (0.442) | 0.373  (0.484) | 0.693  (0.462) | 0.827  (0.379) | 0.959  (0.2) |
| Survival: meso+iodosulfuron (43.2 g ha^-1^) | 0.814  (0.39) | 0.799  (0.401) | 0.754  (0.431) | 0.631  (0.483) | 0.631  (0.483) | 0.0939  (0.292) | 0.445  (0.498) | 0.722  (0.449) | 0.819  (0.386) |
| Biomass: meso+iodosulfuron (mg, 14.4 g ha^-1^) | 526  (452) | 555  (423) | 640  (453) | 399  (372) | 432  (404) | 97  (157) | 297  (366) | 381  (356) | 562  (417) |
| Biomass: meso+iodosulfuron (mg, 43.2 g ha^-1^) | 321  (336) | 312  (343) | 365  (436) | 241  (280) | 323  (364) | 47  (59.2) | 156  (236) | 287  (339) | 372  (342) |
| Survival: fenoxaprop (69 g ha^-1^) | 0.988  (0.11) | 0.974  (0.161) | 0.95  (0.219) | 0.855  (0.353) | 0.955  (0.208) | 0.997  (0.0577) | 0.99  (0.102) | 0.939  (0.241) | 0.953  (0.213) |
| Survival: fenoxaprop (207 g ha^-1^) | 0.969  (0.172) | 0.921  (0.27) | 0.859  (0.349) | 0.763  (0.426) | 0.901  (0.3) | 0.978  (0.148) | 0.907  (0.292) | 0.849  (0.359) | 0.931  (0.254) |
| Biomass: fenoxaprop (mg, 69 g ha^-1^) | 748  (445) | 658  (416) | 670  (437) | 573  (438) | 741  (374) | 722  (348) | 731  (363) | 753  (423) | 737  (419) |
| Biomass: fenoxaprop (mg, 207 g ha^-1^) | 705  (417) | 560  (421) | 561  (434) | 431  (390) | 660  (398) | 658  (356) | 648  (407) | 655  (446) | 663  (447) |
| Biomass: unsprayed (mg) | 762  (468) | 778  (371) | 832  (438) | 847  (399) | 857  (348) | 765  (349) | 870  (390) | 857  (345) | 889  (418) |
